# Growth Differentiation Factor 15 Is Associated With Alzheimer’s Disease Risk

**DOI:** 10.3389/fgene.2021.700371

**Published:** 2021-08-13

**Authors:** Peng-Fei Wu, Xing-Hao Zhang, Ping Zhou, Rui Yin, Xiao-Ting Zhou, Wan Zhang

**Affiliations:** ^1^Hunan Key Laboratory of Animal Models for Human Diseases, Department of Laboratory Animals, Third Xiangya Hospital, Central South University, Changsha, China; ^2^Hunan Key Laboratory of Medical Genetics, Center for Medical Genetics, School of Life Sciences, Central South University, Changsha, China; ^3^Department of Neurology, Beth Israel Deaconess Medical Center, Harvard Medical School, Boston, MA, United States; ^4^Department of Ultrasound, Third Xiangya Hospital, Central South University, Changsha, China; ^5^Department of Biomedical Informatics, Harvard Medical School, Boston, MA, United States; ^6^Department of Neurology, Xiangya Hospital, Central South University, Changsha, China; ^7^Department of Neurology and Neuroscience, Icahn School of Medicine at Mount Sinai, Friedman Brain Institute, New York, NY, United States; ^8^Department of Biology, College of Arts and Sciences, Boston University, Boston, MA, United States

**Keywords:** Alzheimer’s disease, growth differentiation factor 15, Parkinson’s disease, amyotrophic lateral sclerosis, mendelian randomization, genetic epidemiology

## Abstract

**Background:**

Previous observational studies have suggested that associations exist between growth differentiation factor 15 (GDF-15) and neurodegenerative diseases. We aimed to investigate the causal relationships between GDF-15 and Alzheimer’s disease (AD), Parkinson’s disease (PD), and amyotrophic lateral sclerosis (ALS).

**Methods:**

Using summary-level datasets from genome-wide association studies of European ancestry, we performed a two-sample Mendelian randomization (MR) study. Genetic variants significantly associated (*p* < 5 × 10^–8^) with GDF-15 were selected as instrumental variables (*n* = 5). An inverse-variance weighted method was implemented as the primary MR approach, while weighted median, MR–Egger, leave-one-out analysis, and Cochran’s *Q*-test were conducted as sensitivity analyses. All analyses were performed using R 3.6.1 with relevant packages.

**Results:**

MR provided evidence for the association of elevated GDF-15 levels with a higher risk of AD (odds ratio = 1.14; 95% confidence interval, 1.04–1.24; *p* = 0.004). In the reverse direction, Mendelian randomization suggested no causal effect of genetically proxied risk of AD on circulating GDF-15 (*p* = 0.450). The causal effects of GDF-15 on PD (*p* = 0.597) or ALS (*p* = 0.120) were not identified, and the MR results likewise did not support the association of genetic liability to PD or ALS with genetically predicted levels of GDF-15. No evident heterogeneity or horizontal pleiotropy was revealed by multiple sensitivity analyses.

**Conclusion:**

We highlighted the role of GDF-15 in AD as altogether a promising diagnostic marker and a therapeutic target.

## Introduction

Alzheimer’s disease (AD), Parkinson’s disease (PD), and amyotrophic lateral sclerosis (ALS) are common neurodegenerative diseases of aging populations, which lead to the growing healthcare burden worldwide. Most clinical trials targeting potential biochemical pathways in AD, PD, and ALS or modifiable lifestyle factors have failed, leaving merely a handful of medications to ease patient suffering partially or transiently so far (Dorst et al., 2018; Piton et al., 2018; de Bie et al., 2020). Researchers are dedicated to seeking treatments to slow the progression or prevent the onset of these diseases. Identifying causal factors, especially biomarkers, provides promising hints at the possibility of effective and efficient intervention targets.

Growth differentiation factor 15 (GDF-15) belongs to the transforming growth factor-β superfamily (Bootcov et al., 1997). GDF-15 is a cytokine produced in response to inflammatory stressors and has been postulated to associate with healthy aging (Conte et al., 2019, 2020a) and age-related diseases (Ban et al., 2017; Nohara et al., 2019; McGrath et al., 2020; Miyaue et al., 2020; Yue et al., 2020). In the central nervous system, GDF-15 can be produced by lesioned neurons and microglial cells and plays regulatory roles in inflammation (Unsicker et al., 2013). Notably, the role of microglial cells and neuroinflammation in neurodegenerative diseases, especially AD, has been extensively reported (Bartels et al., 2020; Leng and Edison, 2021). Several functional studies (Kim et al., 2015, 2018) reported that GDF-15 was implicated in the Aβ clearance ability of microglial cells. GDF-15 might be linked with neurodegeneration and affects the risk of neurodegenerative diseases.

Previous clinical studies (Chai et al., 2016; McGrath et al., 2020) have also suggested the association of circulating GDF-15 with AD. From a recent prospective study (McGrath et al., 2020), higher circulating GDF-15 was associated with an increased risk of incident AD (hazard ratio = 1.37; *p* = 0.03), but it should be noted that prior smaller cohorts were prone to confounding bias (i.e., healthy conditions and socioeconomic status), and validation of their findings in a larger sample size is required. Studies exploring the relationship between GDF-15 and PD (Davis et al., 2020; Miyaue et al., 2020) and ALS (Ban et al., 2017; Nohara et al., 2019; Yue et al., 2020) likewise failed to draw concrete conclusions. Triangulating evidence across studies with different designs and an augmented sample size is essential to elucidate the role of GDF-15 in AD, PD, and ALS.

Mendelian randomization (MR) is emergingly utilized as an efficient tool to explore the causal relationships between a wide spectrum of exposures, including serum biomarkers, and diseases of interest (Hemani et al., 2018; Liu et al., 2019; He et al., 2020; Zhang et al., 2020). MR is not susceptible to unmeasured confounding factors as traditional observational studies are and has the advantage of overcoming issues of reverse causation (Walker et al., 2019). Single-nucleotide polymorphisms (SNPs) associated with serum GDF-15 levels reaching genome-wide significance (*P* < 5 × 10^–8^) have been reported (Jiang et al., 2018). These SNPs can be employed as genetic instruments, and corresponding association data from genome-wide association studies (GWAS) of AD (Kunkle et al., 2019), PD (Nalls et al., 2019), and ALS (Nicolas et al., 2018) are accessible. Here we conducted a two-sample MR study to explore the causal relationship between GDF-15 and AD, PD, and ALS.

## Materials and Methods

**FIGURE 1:**
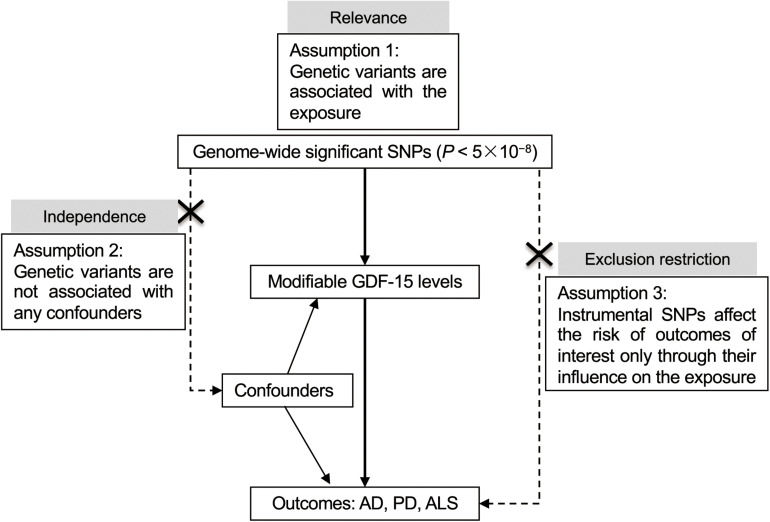
Schematic of the Mendelian randomization study. Three key assumptions underlie the MR study. For illustration, when exploring the causal effect of GDF-15 on AD, genome-wide significant SNPs (*P* < 5 × 10^– 8^) associated with GDF-15 were firstly collected, namely, the relevance assumption. Second, the independence assumption is inherently met given the MR design. Randomized allocation of risk alleles occurs in gamete formation, which far precedes the onset of AD, and is not susceptible to intervening factors in later life as commonly exist in the traditional observational studies. Third, instrumental variables exert their influence on AD only *via* the GDF-15 pathway, known as the exclusion–restriction assumption. MR sensitivity analyses were performed to examine horizontal pleiotropic effects and validate the robustness of the overall estimate. AD, Alzheimer’s disease; ALS, amyotrophic lateral sclerosis; GDF-15, growth differentiation factor 15; MR, Mendelian randomization; PD, Parkinson’s disease; SNP, single-nucleotide polymorphism.

This study was based on publicly accessible datasets, with the informed consent from the participants having been completed by the original study investigators. A schematic of the MR design is presented in Figure 1.

### Instrumental Variables for GDF-15

Summary association statistics for GDF-15 serum concentrations were retrieved from one GWAS (Jiang et al., 2018) conducted in a European population. Four original cohorts underlying the meta-analysis incorporated the Framingham Offspring Cohort (Ho et al., 2012), the Vasculature in Uppsala Seniors Study (Lind et al., 2009), the Northern Sweden Population Health Study (Ek et al., 2016), and the Sydney Memory and Aging Study (Sachdev et al., 2010). About half of the sample of the GWAS (Jiang et al., 2018) consisted of participants from the Framingham Offspring Cohort (*n* = 2,796). The other three cohorts were comprised of randomly recruited community-dwelling participants (∼900 in each cohort). In total, the meta-analysis comprised 5,440 participants with a mean age of 62.1 years, among which 53.1% were female (Supplementary Table 1). The inverse normal transformation was applied, and one standard deviation (SD) of GDF-15 blood levels was approximated as 625.0 pg/ml using immunoassays as previously described (Jiang et al., 2015). Five SNPs reaching genome-wide significance (*P* < 5 × 10^–8^) were kept as instrumental variables (Supplementary Table 2). The effect size (*Beta*) was presented in a unit of SD change in GDF-15 levels per additional effect allele. In the reverse MR investigating whether there were causal effects of neurodegenerative diseases on circulating GDF-15 levels (Sinnott-Armstrong et al., 2021), genetic association data were summarized in Supplementary Table 3.

### Summary Statistics of AD

Genetic associations with AD were retrieved from the recent GWAS by the International Genomics of Alzheimer’s Project (Kunkle et al., 2019). This study included 46 case–control cohorts into the discovery sample, which consisted of 21,982 clinically diagnosed cases and 41,944 controls. The mean ages of cases and controls were 72.9 and 72.4 years, respectively, while the percentages of females were 61.3 and 57.1%, respectively. All participants were non-Hispanic Whites. After quality control and imputation with the European reference panel, 1,000 Genomes Project (Genomes Project Consortium, Auton et al., 2015), the meta-analysis incorporated 9,456,058 common variants (minor allele frequency ≥ 0.01) and 2,024,574 rare variants. The cases were restricted to late-onset AD (onset age > 65 years) as diagnosed by neurologists. The controls were all cognitively normal, excluding mild cognitive impairment. In total, 21 genome-wide significant variants (*P* ≤5 × 10^–8^) were reported and further utilized as instrumental SNPs (Supplementary Table 3) when examining the effect of genetically determined liability to AD on circulating GDF-15 levels.

### Summary Statistics of PD

The summary-level association data for PD were obtained from the largest GWAS by the International Parkinson’s Disease Genomics Consortium (Nalls et al., 2019). There were 17 datasets in the European ancestry meta-analysis, among which summary statistics for 33,674 cases and 449,056 controls were publicly accessible (excluding the 23andMe cohort). The PD cases incorporated both clinically ascertained cases and parental proxy cases from the UK Biobank since self-reported parental history for case ascertainment has been shown as a valid proxy for genetic studies PD in the UK Biobank (Liu et al., 2017). The discovery stage included ∼7.8 million SNPs after routine quality control, from which we retrieved corresponding genetic association data for instrumental SNPs of GDF-15 (Supplementary Table 2) and selected 23 independent SNPs (*P* ≤ 5 × 10^–8^) as proxies for PD in the MR analysis of genetically predicted risk of PD on circulating GDF-15 (Supplementary Table 3).

### Summary Statistics of ALS

Summary statistics were obtained from a large-scale GWAS involving 20,806 ALS cases and 59,804 controls (Nicolas et al., 2018). The dataset was downloaded from ALS Variant Server.^1^ The mean ages of cases and controls were 59.8 and 63.4 years, respectively, while the percentages of females were 41.7 and 69.6%, respectively. The cases were confirmed according to the El Escorial criteria (Hardiman, 2021) by neurologists specializing in ALS, whereas neurologically normal controls were of non-Hispanic white ethnicity mainly from the database of genotypes and phenotypes of NCBI (73.6%). From the final meta-analysis, with 10,031,630 variants included, we obtained six instrumental SNPs associated with ALS at a genome-wide significance level (*P* ≤ 5 × 10^–8^) to investigate the effect of genetically proxied liability to ALS on circulating GDF-15 (Supplementary Table 3).

### Statistical Analysis

MR analyses were implemented using the R language, version 3.6.1 (R Foundation for Statistical Computing, Vienna, Austria), with the TwoSampleMR (version 0.5.3) package (Hemani et al., 2018; Walker et al., 2019) and the MendelianRandomization (version 0.5.0) package (Broadbent et al., 2020). For each instrumental variable, the Wald ratio was calculated by dividing the SNP outcome association by the SNP exposure coefficient. Given the SNP exposure effect size *X_k_* and its standard error σ*X_k_* and the SNP outcome statistics *Y_k_* and σ*Y*_*k*_, the MR estimate can be derived by the Wald ratio *Y*_*k*_/*X*_*k*_ with its standard error σ*Y*_*k*_/*X*_*k*_. Then, the primary MR method, the inverse-variance weighted (IVW) model (Burgess et al., 2013), gave an overall estimate ${\hat{\beta }}_{\text{MR}}$ and its standard error ${\hat{\sigma }}_{\text{MR}}$ using the formula: ${\hat{\beta }}_{\text{MR}}$ and ${\hat{\sigma }}_{\text{MR}}=\sqrt{1/\sum X_{k}^{2}\,\sigma_{Y_{k}}^{-2}}$. The *r*-value matrix which measured the$=\sum X_{k}\,Y_{k}\,\sigma_{Y_{k}}^{-2}/\sum X_{k}^{2}\,\sigma_{Y_{k}}^{-2}$ linkage disequilibrium between instrumental SNPs for GDF-15 (Supplementary Table 4) was input into the modified IVW model (Burgess et al., 2016) to generate a causal estimate adjusted for correlations between instrumental SNPs. The IVW estimates would be biased if not all variants are valid or if an unbalanced pleiotropy exists (Burgess et al., 2019), and hence additional MR methods were also implemented. The weighted median estimator pooled effects of individual variants efficiently under the prerequisite that more than 50% of the weight came from valid instrumental variables (Bowden et al., 2016). MR–Egger methods were capable of identifying pleiotropic effects and yielding overall estimates (MR–Egger slope) adjusted for them (Bowden et al., 2015), whereas the MR–Egger intercept could be used to measure the unbalanced horizontal pleiotropy. We further performed leave-one-out analysis, and *I*^2^ statistics given by Cochran’s *Q*-test were employed to evaluate heterogeneous effects within instrumental variants (Greco et al., 2015; Bowden et al., 2018). A posterior power calculation was performed using mRnd (Brion et al., 2013) at the given significance level of 0.05 and statistical power of 80%. Forest plots and scatter plots were generated to visualize overall causal estimates, where the odds ratio (OR) and 95% confidence interval (CI) represented the risk of AD, PD, or ALS per one SD increase in circulating GDF-15 concentrations, and the Beta and 95% CI represented a change in GDF-15 per one-unit increase in the log-OR of neurodegenerative diseases in the bidirectional MR. The significance threshold was *P* < 0.05/6, corrected by the Bonferroni method.

## Results

### Primary MR Investigating the Effect of GDF-15 on AD

**FIGURE 2:**
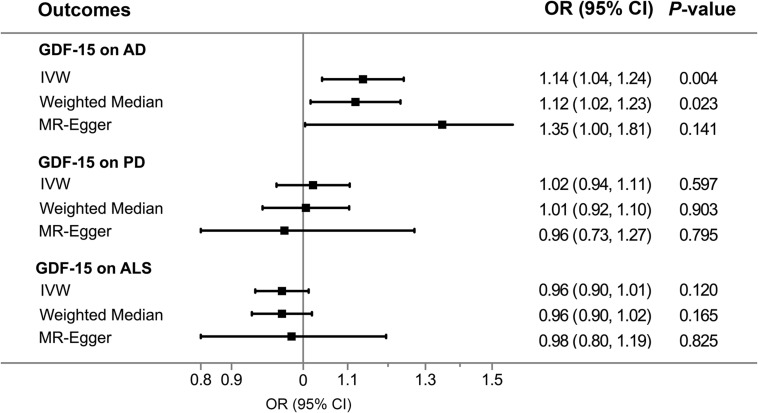
Association of genetically predicted GDF-15 with three neurodegenerative diseases by Mendelian randomization approaches. Elevated levels of circulating GDF-15 were associated with a higher AD risk (OR = 1.14; 95% CI, 1.04–1.24; *P* = 0.004) per one SD increase in GDF-15, whereas there were no causal effects of GDF-15 on PD and ALS. AD, Alzheimer’s disease; ALS, amyotrophic lateral sclerosis; CI, confidence interval; GDF-15, growth differentiation factor 15; IVW, inverse-variance weighted model; OR, odds ratio; PD, Parkinson’s disease.

**FIGURE 3:**
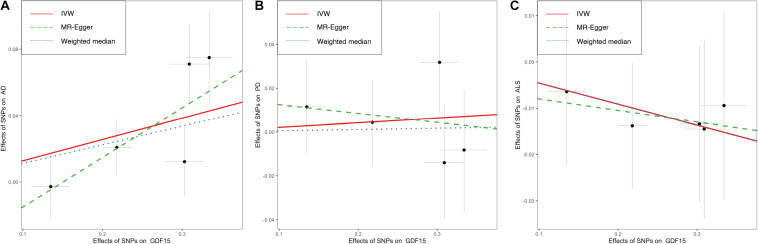
Scatter plots showing the effects of GDF-15 on three neurodegenerative diseases. No evident outlier SNPs were present in the Mendelian randomization analyses of GDF-15 on the risk of Alzheimer’s disease **(A)**, Parkinson’s disease **(B)**, and amyotrophic lateral sclerosis **(C)**. As delineated by fitted lines, overall causal estimates were given by the IVW method (red solid line), weighted median (blue dotted line), and MR–Egger slope (green dashed lines). Individual SNP (black point) with its effect on the risk of neurodegenerative diseases (vertical line) against its effect on circulating levels of GDF-15 (horizontal line) was depicted in the background. AD, Alzheimer’s disease; ALS, amyotrophic lateral sclerosis; CI, confidence interval; GDF-15, growth differentiation factor 15; IVW, inverse-variance weighted model; PD, Parkinson’s disease; SNP, single-nucleotide polymorphism.

**FIGURE 4:**
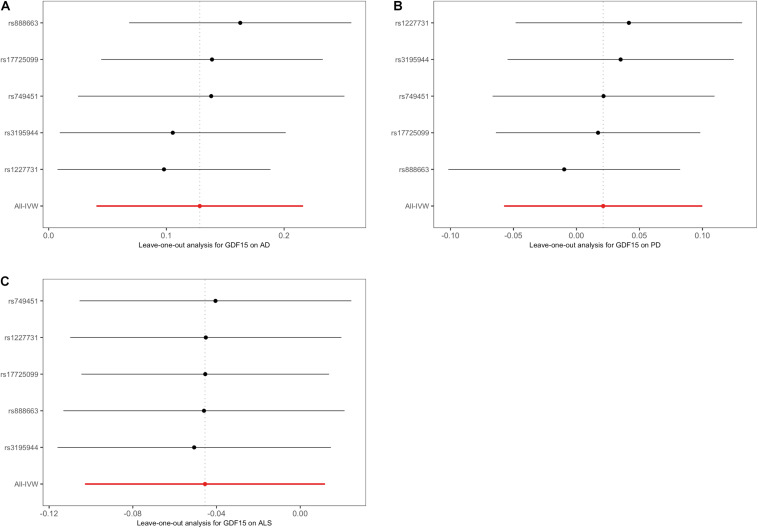
Leave-one-out plots in the Mendelian randomization sensitivity analyses of GDF-15 on three neurodegenerative diseases. There was no evidence of obvious heterogeneity, indicating that no specific SNP alone accounted for the association of GDF-15 with Alzheimer’s disease **(A)**, Parkinson’s disease **(B)**, and amyotrophic lateral sclerosis **(C)**. The IVW causal estimate and how the overall estimate (red horizontal line) was disproportionately driven, which is influenced by the removal of a single variant (black horizontal line), were visualized. AD, Alzheimer’s disease; ALS, amyotrophic lateral sclerosis; GDF-15, growth differentiation factor 15; IVW, inverse-variance weighted model; PD, Parkinson’s disease.

Overall, The MR results suggested that elevated serum GDF-15 levels were associated with a higher AD risk. By the IVW method (Figure 2), the OR of AD was 1.14 (95% CI, 1.04–1.24; *p* = 0.004) per one SD increment in genetically predicted concentrations of GDF-15. The weighted median estimator also provided suggestive evidence for the relationship (OR = 1.12; 95% CI, 1.02–1.23; *p* = 0.023) but failed to reach Bonferroni significance. Notably, Cochran’s *Q*-test (*I*^2^ = 32.5%, *p* = 0.205) and MR–Egger regression (intercept = −0.045, *p* = 0.323) indicated no existence of pleiotropic or outlying variant which would drive the overall estimate disproportionately. In this case, weighted median and MR–Egger regression were not likely to generate as precise estimates as the IVW approach. According to our power estimation, given the variance of GDF-15 explained by instrumental variables of 21.5%, the MR analysis has sufficient power (> 80%) to detect a large effect size (OR > 1.057 or OR < 0.945). Although no heterogenous variants were revealed in the scatter plot (Figure 3) and leave-one-out analysis (Figure 4), the causal estimates given by Wald ratios from both rs3195944 (OR = 1.25; 95% CI, 1.06–1.48; *p* = 0.007) and rs1227731 (OR = 1.26; 95% CI, 1.08–1.46; *p* = 0.003) were notably nominally significant (*p* < 0.05) when examining the effect of GDF-15 on AD mediated by individual SNP (Supplementary Table 5). After searching the Ensemble and dbSNP databases, rs3195944 (chr19:18476711) was located in the last exon (3′ UTR) of *PGPEP1* (transcript NM_017712, c.^∗^2318A > G), and rs1227731 (chr19:18497903) was located in the intron between the only two exons of *GDF15* (transcript NM_004864, 627 bp downstream of exon 1 and 1,192 bp upstream of exon 2). Albeit in non-coding regions, given the proximity and the specificity of their locations (Supplementary Figure 1), both variants were candidates to regulate the transcription of the closest genes and hence contribute to the mechanism of effect for circulating GDF-15 on AD risk.

### Reverse MR Exploring the Association of AD With GDF-15

In the reverse direction, MR analyses (Supplementary Figure 2) did not support the causal effect of the genetically determined risk of AD on circulating levels of GDF-15. A one-unit increase in log-OR of AD was associated with 0.011 SD change in circulating GDF-15 (95% CI, −0.018to 0.041; *p* = 0.450) by the IVW method. Scatter plots (Supplementary Figure 3) and leave-one-out plots (Supplementary Figure 4) suggested that no specific variant drove the overall estimate disproportionately. The MR–Egger intercept (intercept = 0.006, *p* = 0.202) and Cochran’s *Q*-test (*I*^2^ = 21.3%, *p* = 0.186) indicated no existence of horizontal pleiotropy or obvious heterogeneity.

### GDF-15 in Relation to PD and ALS

The MR analyses did not support the relationship between GDF-15 and PD or ALS (Figure 2). A change in the concentrations of serum GDF-15 was not associated with the risk of PD (OR = 1.02; 95% CI, 0.94–1.11), as the estimate was not statistically significant (*p* = 0.597). There was likewise no causal effect of GDF-15 on the risk of ALS (OR = 0.96; 95% CI, 0.90–1.01; *p* = 0.120) per one-SD increase in GDF-15 levels. Additional MR methods provided consistent results. Furthermore, no heterogeneity was identified in the MR analysis on PD (*Q* = 2.27, *p* = 0.685) or ALS (*Q* = 0.16, *p* = 0.997), and no pleiotropy was detected for PD (intercept = 0.016, *p* = 0.683) or ALS (intercept = −0.005, *p* = 0.849). Nevertheless, this study would be underpowered to detect the OR intervals of 0.966–1.034 for PD and 0.952–1.049 for ALS.

The reverse MR exploring the effect of genetically predicted liability to PD or ALS on circulating GDF-15 (Supplementary Figure 2) did not provide statistically significant evidence for the causal relationships. The change in circulating GDF-15 was 0.028 SD (95% CI, −0.001 to 0.058; *p* = 0.062) per one-unit increase in log-OR of PD, whereas it was −0.002 SD (95% CI, −0.078 to 0.075; *p* = 0.966) for ALS. Multiple sensitivity analyses (Supplementary Figures 3, 4 and Supplementary Table 6) demonstrated no evident heterogeneity or pleiotropy and suggested the general robustness of IVW estimates for the association of PD and ALS with GDF-15.

## Discussion

Our study found that genetically predicted circulating GDF-15 was associated with the risk of AD, but not PD or ALS. To the best of our knowledge, no studies before have employed MR to investigate the association of GDF-15 with neurodegenerative diseases. Several observational studies (Kim et al., 2015; Conte et al., 2020b; McGrath et al., 2020) have tried to establish the relationship between serum GDF-15 and AD. One study conducted in a dementia-free Framingham cohort, among 1,603 participants of European ancestry with a median of 11.8-year follow-up (McGrath et al., 2020), suggested that elevated GDF-15 was associated with a higher risk of AD (hazard ratio = 1.37 per SD increase in natural log-transformed value; 95% CI, 1.03–1.81; *p* = 0.03), implicating its predictive value as a novel AD biomarker. Another recent study (Conte et al., 2020b) among 120 Italian AD patients did not observe a difference in the plasma levels of GDF-15 in comparison to healthy aging controls; nevertheless, this study confirmed its positive correlation with age in healthy participants, indicating a possible role of GDF-15 in normal aging. Studies in other populations are scarce. Chai et al. (2016) identified an association of GDF-15 with AD in Southeast Asians. To be specific, using a tertile categorization with two cut-points of 902 and 1,563 pg/ml, the relationship between GDF-15 and AD (OR = 4.93; 95% CI, 1.31–18.61) in the highest (third) tertile was reported with the lowest tertile as the reference group. These cohort or case–control studies are notably prone to confounding bias and limited by sample size and cannot establish the causal role of GDF-15 in AD convincingly.

In addition to clinical studies, researchers (Kim et al., 2015, 2018) have explored the potential mechanism implicated in the effect of GDF-15 on AD. Using human umbilical cord blood-derived mesenchymal stem cells (Kim et al., 2015), GDF-15 was found to be associated with the proliferation of neural stem cells and neuronal differentiation and involved in hippocampal neurogenesis. Furthermore, using the 5XFAD mouse model (Kim et al., 2018), GDF-15 was implicated in Aβ clearance by microglia cells, and GDF-15 affected the expression of *IDE* (encoding the protein Insulin-Degrading Enzyme, IDE). TGFβRII (TGFβ receptor type II), which is mainly expressed in microglia and neurons, serves as the mediator and receptor and bridges the effect of GDF-15 on IDE. IDE is required downstream for Aβ degradation in neurons and microglial cells (Pivovarova et al., 2016) and hence plays a role in the pathogenesis of AD. The pathway from GDF-15 to TGFβRII to IDE as a potential therapeutic target for AD warrants further investigation.

As for the relationship between GDF-15 and PD, elevated levels of GDF-15 were observed in PD patients compared to age-matched controls (1,472 vs. 1,093 pg/ml, *p* = 0.034) in a Japanese cohort (Miyaue et al., 2020). Similarly, in Chinese Han populations, an earlier study (Yao et al., 2017) reported the difference and found that GDF-15 was an independent risk factor for the Unified PD Rating Scale-III score, further suggesting its association with disease severity. As for the participants of European ancestry, however, one recent study (Davis et al., 2020) failed to identify GDF-15 as a biomarker for PD. One reason may be that these studies had relatively small sample sizes of participants. Another reason is that population differences between Europeans and Asians might exist.

There have not been enough studies (Nohara et al., 2019; Straub et al., 2021) exploring the association of GDF-15 with ALS so far. In a Japanese cohort (Nohara et al., 2019) aimed at studying the effect of GDF-15 on mitochondrial diseases, GDF-15 was positively correlated with age (*r* = 0.72, *p* < 0.001) in ALS patients. One recent study (Straub et al., 2021) investigating the function of *CHCHD10* in ALS demonstrated that GDF-15 was involved in the activation of the mitochondrial unfolded protein response, which might contribute to the selective vulnerability of motor neurons in the pathogenesis of ALS. More studies are necessary to elucidate the relationship between GDF-15 and PD and ALS.

The primary strength of this study lies in the application of two-sample MR, which has benefits in circumventing residual confounding, avoiding reverse causation, and enhancing power by a large sample size. There are several limitations as well. Firstly, genetic variant-proxied GDF-15 levels represent overall serum levels across the lifespan. We should be cautious with the effect estimate in regards to translational medicine and pharmaceutical development, even if GDF-15 has the potential to be a target, since drug treatment generally takes effect in a smooth and transient fashion. The finding was also based on European-ancestry datasets, and the participants in the GWAS of GDF-15 were from four community-based cohorts, whereas the participants in the GWAS of AD were from a much larger population; great caution should be exercised in the interpretation and generalization.

In conclusion, we highlighted the role of GDF-15 in AD as a promising diagnostic marker and therapeutic target. Triangulating evidence across clinical observational, genetic epidemiological, and biological mechanistic studies is warranted to shed light on whether targeting GDF-15 is an effective treatment for AD, PD, and ALS.

## Data Availability Statement

The original contributions presented in the study are included in the article/Supplementary Material, further inquiries can be directed to the corresponding author/s.

## Author Contributions

P-FW, X-HZ, and PZ contributed to the conception and design of the study. P-FW, X-HZ, RY, and X-TZ contributed to the acquisition and analysis of data. P-FW, X-HZ, and WZ contributed to the drafting and review of the main manuscript, tables, and figures. All authors contributed to the article and approved the submitted version.

## Conflict of Interest

The authors declare that the research was conducted in the absence of any commercial or financial relationships that could be construed as a potential conflict of interest.

## Publisher’s Note

All claims expressed in this article are solely those of the authors and do not necessarily represent those of their affiliated organizations, or those of the publisher, the editors and the reviewers. Any product that may be evaluated in this article, or claim that may be made by its manufacturer, is not guaranteed or endorsed by the publisher.

## Footnotes

1
http://als.umassmed.edu/

## References

[B1] Ban N. Siegfried C. J. Lin J. B. Shui Y. B. Sein J. Pita-Thomas W. (2017). GDF15 is elevated in mice following retinal ganglion cell death and in glaucoma patients. *JCI Insight* 2:e91455. 10.1172/jci.insight.91455 28469085 PMC5414567

[B2] Bartels T. De Schepper S. Hong S. (2020). Microglia modulate neurodegeneration in Alzheimer’s and Parkinson’s diseases. *Science* 370 66–69. 10.1126/science.abb8587 33004513

[B3] Bootcov M. R. Bauskin A. R. Valenzuela S. M. Moore A. G. Bansal M. He X. Y. (1997). MIC-1, a novel macrophage inhibitory cytokine, is a divergent member of the TGF-beta superfamily. *Proc. Natl. Acad. Sci. U. S. A.* 94 11514–11519. 10.1073/pnas.94.21.11514 9326641 PMC23523

[B4] Bowden J. Davey Smith G. Burgess S. (2015). Mendelian randomization with invalid instruments: effect estimation and bias detection through Egger regression. *Int. J. Epidemiol.* 44 512–525. 10.1093/ije/dyv080 26050253 PMC4469799

[B5] Bowden J. Davey Smith G. Haycock P. C. Burgess S. (2016). Consistent estimation in Mendelian randomization with some invalid instruments using a weighted median estimator. *Genet. Epidemiol.* 40 304–314. 10.1002/gepi.21965 27061298 PMC4849733

[B6] Bowden J. Hemani G. Davey Smith G. (2018). Invited commentary: detecting individual and global horizontal pleiotropy in Mendelian randomization-a job for the humble heterogeneity statistic? *Am. J. Epidemiol.* 187 2681–2685. 10.1093/aje/kwy185 30188969 PMC6269239

[B7] Brion M. J. Shakhbazov K. Visscher P. M. (2013). Calculating statistical power in Mendelian randomization studies. *Int. J. Epidemiol.* 42 1497–1501. 10.1093/ije/dyt179 24159078 PMC3807619

[B8] Broadbent J. R. Foley C. N. Grant A. J. Mason A. M. Staley J. R. Burgess S. (2020). MendelianRandomization v0.5.0: updates to an R package for performing Mendelian randomization analyses using summarized data. *Wellcome Open Res.* 5:252. 10.12688/wellcomeopenres.16374.2 33381656 PMC7745186

[B9] Burgess S. Butterworth A. Thompson S. G. (2013). Mendelian randomization analysis with multiple genetic variants using summarized data. *Genet. Epidemiol.* 37 658–665. 10.1002/gepi.21758 24114802 PMC4377079

[B10] Burgess S. Davey Smith G. Davies N. M. Dudbridge F. Gill D. Glymour M. M. (2019). Guidelines for performing Mendelian randomization investigations. *Wellcome Open Res.* 4:186. 10.12688/wellcomeopenres.15555.2 32760811 PMC7384151

[B11] Burgess S. Dudbridge F. Thompson S. G. (2016). Combining information on multiple instrumental variables in Mendelian randomization: comparison of allele score and summarized data methods. *Stat. Med.* 35 1880–1906. 10.1002/sim.6835 26661904 PMC4832315

[B12] Chai Y. L. Hilal S. Chong J. P. C. Ng Y. X. Liew O. W. Xu X. (2016). Growth differentiation factor-15 and white matter hyperintensities in cognitive impairment and dementia. *Medicine (Baltimore)* 95:e4566. 10.1097/MD.0000000000004566 27537582 PMC5370808

[B13] Conte M. Martucci M. Mosconi G. Chiariello A. Cappuccilli M. Totti V. (2020a). GDF15 plasma level is inversely associated with level of physical activity and correlates with markers of inflammation and muscle weakness. *Front. Immunol.* 11:915. 10.3389/fimmu.2020.00915 32477368 PMC7235447

[B14] Conte M. Ostan R. Fabbri C. Santoro A. Guidarelli G. Vitale G. (2019). Human aging and longevity are characterized by high levels of mitokines. *J. Gerontol. A Biol. Sci. Med. Sci.* 74 600–607. 10.1093/gerona/gly153 29955888

[B15] Conte M. Sabbatinelli J. Chiariello A. Martucci M. Santoro A. Monti D. (2020b). Disease-specific plasma levels of mitokines FGF21, GDF15, and humanin in type II diabetes and Alzheimer’s disease in comparison with healthy aging. *Geroscience* 43 985–1001. 10.1007/s11357-020-00287-w 33131010 PMC8110619

[B16] Davis R. L. Wong S. L. Carling P. J. Payne T. Sue C. M. Bandmann O. (2020). Serum FGF-21, GDF-15, and blood mtDNA copy number are not biomarkers of Parkinson disease. *Neurol. Clin. Pract.* 10 40–46. 10.1212/CPJ.0000000000000702 32190419 PMC7057070

[B17] de Bie R. M. A. Clarke C. E. Espay A. J. Fox S. H. Lang A. E. (2020). Initiation of pharmacological therapy in Parkinson’s disease: when, why, and how. *Lancet Neurol.* 19 452–461. 10.1016/S1474-4422(20)30036-3 32171387

[B18] Dorst J. Ludolph A. C. Huebers A. (2018). Disease-modifying and symptomatic treatment of amyotrophic lateral sclerosis. *Ther. Adv. Neurol. Disord.* 11:1756285617734734. 10.1177/1756285617734734 29399045 PMC5784546

[B19] Ek W. E. Hedman A. K. Enroth S. Morris A. P. Lindgren C. M. Mahajan A. (2016). Genome-wide DNA methylation study identifies genes associated with the cardiovascular biomarker GDF-15. *Hum. Mol. Genet.* 25 817–827. 10.1093/hmg/ddv511 26681806 PMC5557339

[B20] Genomes Project Consortium, Auton A. Brooks L. D. Durbin R. M. Garrison E. P. Kang H. M. (2015). A global reference for human genetic variation. *Nature* 526 68–74. 10.1038/nature15393 26432245 PMC4750478

[B21] Greco M. F. Minelli C. Sheehan N. A. Thompson J. R. (2015). Detecting pleiotropy in Mendelian randomisation studies with summary data and a continuous outcome. *Stat. Med.* 34 2926–2940. 10.1002/sim.6522 25950993

[B22] Hardiman O. (2021). Major advances in amyotrophic lateral sclerosis in 2020. *Lancet Neurol.* 20 14–15. 10.1016/S1474-4422(20)30447-6 33340474

[B23] He Y. Zhang H. Wang T. Han Z. Ni Q. B. Wang K. (2020). Impact of serum calcium levels on Alzheimer’s disease: a Mendelian randomization study. *J. Alzheimers Dis.* 76 713–724. 10.3233/JAD-191249 32538835

[B24] Hemani G. Zheng J. Elsworth B. Wade K. H. Haberland V. Baird D. (2018). The MR-base platform supports systematic causal inference across the human phenome. *Elife* 7:e34408. 10.7554/eLife.34408 29846171 PMC5976434

[B25] Ho J. E. Mahajan A. Chen M. H. Larson M. G. Mccabe E. L. Ghorbani A. (2012). Clinical and genetic correlates of growth differentiation factor 15 in the community. *Clin. Chem.* 58 1582–1591. 10.1373/clinchem.2012.190322 22997280 PMC4150608

[B26] Jiang J. Thalamuthu A. Ho J. E. Mahajan A. Ek W. E. Brown D. A. (2018). A Meta-analysis of genome-wide association studies of growth differentiation factor-15 concentration in blood. *Front. Genet.* 9:97. 10.3389/fgene.2018.00097 29628937 PMC5876753

[B27] Jiang J. Wen W. Brown D. A. Crawford J. Thalamuthu A. Smith E. (2015). The relationship of serum macrophage inhibitory cytokine-1 levels with gray matter volumes in community-dwelling older individuals. *PLoS One* 10:e0123399. 10.1371/journal.pone.0123399 25867953 PMC4395016

[B28] Kim D. H. Lee D. Chang E. H. Kim J. H. Hwang J. W. Kim J. Y. (2015). GDF-15 secreted from human umbilical cord blood mesenchymal stem cells delivered through the cerebrospinal fluid promotes hippocampal neurogenesis and synaptic activity in an Alzheimer’s disease model. *Stem Cells Dev.* 24 2378–2390. 10.1089/scd.2014.0487 26154268 PMC4598918

[B29] Kim D. H. Lee D. Lim H. Choi S. J. Oh W. Yang Y. S. (2018). Effect of growth differentiation factor-15 secreted by human umbilical cord blood-derived mesenchymal stem cells on amyloid beta levels in in vitro and in vivo models of Alzheimer’s disease. *Biochem. Biophys. Res. Commun.* 504 933–940. 10.1016/j.bbrc.2018.09.012 30224067

[B30] Kunkle B. W. Grenier-Boley B. Sims R. Bis J. C. Damotte V. Naj A. C. (2019). Genetic meta-analysis of diagnosed Alzheimer’s disease identifies new risk loci and implicates Abeta, tau, immunity and lipid processing. *Nat. Genet.* 51 414–430. 30820047 10.1038/s41588-019-0358-2 PMC6463297

[B31] Leng F. Edison P. (2021). Neuroinflammation and microglial activation in Alzheimer disease: where do we go from here? *Nat. Rev. Neurol.* 17 157–172. 10.1038/s41582-020-00435-y 33318676

[B32] Lind L. Wallentin L. Kempf T. Tapken H. Quint A. Lindahl B. (2009). Growth-differentiation factor-15 is an independent marker of cardiovascular dysfunction and disease in the elderly: results from the prospective investigation of the vasculature in uppsala Seniors (PIVUS) Study. *Eur. Heart J.* 30 2346–2353. 10.1093/eurheartj/ehp261 19561023

[B33] Liu G. Jin S. Jiang Q. (2019). Interleukin-6 receptor and inflammatory bowel disease: a Mendelian randomization study. *Gastroenterology* 156 823–824. 10.1053/j.gastro.2018.09.059 30445015

[B34] Liu J. Z. Erlich Y. Pickrell J. K. (2017). Case-control association mapping by proxy using family history of disease. *Nat. Genet.* 49 325–331. 10.1038/ng.3766 28092683

[B35] McGrath E. R. Himali J. J. Levy D. Conner S. C. DeCarli C. Pase M. P. (2020). Growth differentiation factor 15 and NT-proBNP as blood-based markers of vascular brain injury and dementia. *J. Am. Heart Assoc.* 9:e014659. 10.1161/JAHA.119.014659 32921207 PMC7792414

[B36] Miyaue N. Yabe H. Nagai M. (2020). Serum growth differentiation factor 15, but not lactate, is elevated in patients with Parkinson’s disease. *J. Neurol. Sci.* 409:116616. 10.1016/j.jns.2019.116616 31862518

[B37] Nalls M. A. Blauwendraat C. Vallerga C. L. Heilbron K. Bandres-Ciga S. Chang D. (2019). Identification of novel risk loci, causal insights, and heritable risk for Parkinson’s disease: a meta-analysis of genome-wide association studies. *Lancet Neurol.* 18 1091–1102. 10.1016/S1474-4422(19)30320-5 31701892 PMC8422160

[B38] Nicolas A. Kenna K. P. Renton A. E. Ticozzi N. Faghri F. Chia R. (2018). Genome-wide analyses identify KIF5A as a novel ALS gene. *Neuron* 97:e1266. 10.1016/j.neuron.2018.02.027 29566793 PMC5867896

[B39] Nohara S. Ishii A. Yamamoto F. Yanagiha K. Moriyama T. Tozaka N. (2019). GDF-15, a mitochondrial disease biomarker, is associated with the severity of multiple sclerosis. *J. Neurol. Sci.* 405:116429. 10.1016/j.jns.2019.116429 31476622

[B40] Piton M. Hirtz C. Desmetz C. Milhau J. Lajoix A. D. Bennys K. (2018). Alzheimer’s disease: advances in drug development. *J. Alzheimers Dis.* 65 3–13. 10.3233/JAD-180145 30040716

[B41] Pivovarova O. Höhn A. Grune T. Pfeiffer A. F. Rudovich N. (2016). Insulin-degrading enzyme: new therapeutic target for diabetes and Alzheimer’s disease? *Ann. Med.* 48 614–624. 10.1080/07853890.2016.1197416 27320287

[B42] Sachdev P. S. Brodaty H. Reppermund S. Kochan N. A. Trollor J. N. Draper B. (2010). The sydney memory and ageing study (MAS): methodology and baseline medical and neuropsychiatric characteristics of an elderly epidemiological non-demented cohort of Australians aged 70-90 years. *Int. Psychogeriatr.* 22 1248–1264. 10.1017/S1041610210001067 20637138

[B43] Sinnott-Armstrong N. Tanigawa Y. Amar D. Mars N. Benner C. Aguirre M. (2021). Genetics of 35 blood and urine biomarkers in the UK Biobank. *Nat. Genet.* 53 185–194. 10.1038/s41588-020-00757-z 33462484 PMC7867639

[B44] Straub I. R. Weraarpachai W. Shoubridge E. A. (2021). Multi-OMICS study of a CHCHD10 variant causing ALS demonstrates metabolic rewiring and activation of endoplasmic reticulum and mitochondrial unfolded protein responses. *Hum. Mol. Genet.* 30 687–705. 10.1093/hmg/ddab078 33749723 PMC8127406

[B45] Unsicker K. Spittau B. Krieglstein K. (2013). The multiple facets of the TGF-beta family cytokine growth/differentiation factor-15/macrophage inhibitory cytokine-1. *Cytokine Growth Factor Rev.* 24 373–384. 10.1016/j.cytogfr.2013.05.003 23787157

[B46] Walker V. M. Davies N. M. Hemani G. Zheng J. Haycock P. C. Gaunt T. R. (2019). Using the MR-Base platform to investigate risk factors and drug targets for thousands of phenotypes. *Wellcome Open Res.* 4:113. 10.12688/wellcomeopenres.15334.2 31448343 PMC6694718

[B47] Yao X. Wang D. Zhang L. Wang L. Zhao Z. Chen S. (2017). Serum growth differentiation factor 15 in Parkinson disease. *Neurodegener. Dis.* 17 251–260. 10.1159/000477349 28787735

[B48] Yue T. Lu H. Yao X. M. Du X. Wang L. L. Guo D. D. (2020). Elevated serum growth differentiation factor 15 in multiple system atrophy patients: a case control study. *World J. Clin. Cases* 8 2473–2483. 10.12998/wjcc.v8.i12.2473 32607324 PMC7322433

[B49] Zhang H. Wang T. Han Z. Liu G. (2020). Mendelian randomization study to evaluate the effects of interleukin-6 signaling on four neurodegenerative diseases. *Neurol. Sci.* 41 2875–2882. 10.1007/s10072-020-04381-x 32328834

