# Supplementary material for: Growth Differentiation Factor 15 Is Associated With Alzheimer’s Disease Risk
Source: Front Genet. 2021 Aug 13;12:700371. doi: 10.3389/fgene.2021.700371 (PMC8414585; doi:10.3389/fgene.2021.700371)
Supplement: Supplementary file 1 [file Data_Sheet_1.PDF]

Supplementary Material

Supplementary Table 1. Source dataset for instrumental variables utilized in the Mendelian randomization analysis…………………2

Supplementary Table 2. Characteristics of genetic instrumental variables in the main Mendelian randomization of GDF-15 on neurodegenerative diseases……………………………………………………………………………………………..3

Supplementary Table 3. Summary statistics utilized in the reverse Mendelian randomization exploring effects of neurodegenerative diseases on GDF-15………………………………………………………………………………………………….4

Supplementary Table 4. Linkage disequilibrium matrix (*r*-value) utilized in the IVW method adjusted for correlated instrumental variables of GDF-15………………………………………………………………………………………………….8

Supplementary Table 5. Associations of GDF-15 with AD, PD and ALS in Mendelian randomization analyses by each instrumental variant and IVW adjusted for correlations……………………………………………………………………………..9

Supplementary Table 6. Mendelian randomization sensitivity analyses examining horizontally pleiotropy and heterogeneity……....11

Supplementary Figure 1. Pairwise statistics for linkage disequilibrium between instrumental variants for GDF-15…………………13

Supplementary Figure 2. Effects of genetically-predicted risk of AD, PD and ALS on circulating GDF-15 by Mendelian randomization analyses………...14

Supplementary Figure 3. Scatter plots showing effects of three neurodegenerative diseases on circulating GDF-15…………………15

Supplementary Figure 4. Leave-one-out plots in the Mendelian randomization sensitivity analyses of AD, PD and ALS on circulating GDF-15………...16

Supplementary Texts and References………………………………………………………………………………17

Supplementary Table 1. Source dataset for instrumental variables utilized in the Mendelian randomization analysis

Trait	Instrumental SNPs	Sample Size (Cases/Controls)	Female (%)	Mean Age (Years)	Adjusted Covariates	Inflation factor λ_{GC}	Effect Unit	Source
GDF-15	5	5440	53.1%	62.1	sex, age, 2 principal components	1.004	SD (625 pg/ml)	Jiang et al., 2018 (1)
AD	21	21,982 / 41,944	58.5%	72.6	sex, age, 5 principal components	1.05	log-odds ratio	Kunkle et al., 2019 (2)
PD	23	33,674 / 449,056	54.0%	56.8	sex, age, 5 principal components	1.17	log-odds ratio	Nalls et al., 2019 (3)
ALS	6	20,806 / 59,804	64.5%	62.7	sex, age, 2 to 4 principal components	0.93	log-odds ratio	Nicolas et al., 2018 (4)

Abbreviations: AD, Alzheimer's disease; ALS, amyotrophic lateral sclerosis; GDF-15, Growth Differentiation Factor-15; PD, Parkinson's disease; SD, standard deviation; SNP, Single-nucleotide polymorphism.

Note: Summary statistics were yielded in additive logistic model adjusting for primary covariates (biological sex, age at onset in cases and age at last examination for controls, principal components underlying population substructure).

Supplementary Table 2. Characteristics of genetic instrumental variables in the main Mendelian randomization of GDF-15 on neurodegenerative diseases

SNP	Coordinate (GRCh37)	EA	OA	EAF	GDF-15		AD		PD		ALS	
					Beta (SE)	*P*-value	Beta (SE)	*P*-value	Beta (SE)	*P*-value	Beta (SE)	*P*-value
rs888663	19:18484922	T	G	0.81	0.303 (0.024)	2.64×10^{-35}	-0.012 (0.021)	0.559	0.032 (0.023)	0.175	-0.013 (0.017)	0.432
rs1227731	19:18497903	A	G	0.69	0.309 (0.026)	3.37×10^{-35}	0.071 (0.024)	0.003	-0.014 (0.026)	0.586	-0.015 (0.019)	0.455
rs749451	19:18479647	C	T	0.57	0.218 (0.019)	2.54×10^{-31}	-0.021 (0.017)	0.211	0.004 (0.019)	0.820	0.014 (0.014)	0.312
rs3195944	19:18476711	G	A	0.12	0.334 (0.029)	2.39×10^{-30}	0.075 (0.028)	0.007	-0.008 (0.028)	0.769	0.009 (0.02)	0.644
rs17725099	19:18482358	A	G	0.24	0.135 (0.025)	4.13×10^{-8}	-0.003 (0.019)	0.891	0.012 (0.022)	0.604	-0.006 (0.016)	0.687

Note: SNP, Single-nucleotide polymorphism; EA, effect allele; OA, other allele; EAF, effect allele frequency; Beta (SE) represents coefficients by the additive regression model, and Beta > 0 denotes an additional copy of effect allele can increase serum GDF-15 levels or risks of AD/PD/ALS.

Supplementary Table 3. Summary statistics utilized in the reverse Mendelian randomization exploring effects of neurodegenerative diseases on GDF-15

Traits	SNP	Coordinate (GRCh37)	EA	OA	Association with exposures			Association with GDF-15		
					Beta	SE	*P*-value	Beta	SE	*P*-value
AD	rs1081105	19:45412955	C	A	0.942	0.044	1.51×10^{-103}	-0.037	0.031	0.230
AD	rs111278137	19:45215081	A	G	-0.474	0.071	3.20×10^{-11}	-0.009	0.046	0.846
AD	rs11257242	10:11721119	G	C	0.084	0.015	4.64×10^{-08}	0.002	0.010	0.828
AD	rs114812713	6:41034000	C	G	0.298	0.043	4.47×10^{-12}	0.062	0.040	0.122
AD	rs11767557	7:143109139	C	T	-0.103	0.018	1.56×10^{-08}	-0.025	0.012	0.036
AD	rs12151021	19:1050874	G	A	-0.107	0.017	2.56×10^{-10}	0.015	0.011	0.152
AD	rs12590654	14:92938855	A	G	-0.091	0.016	8.73×10^{-09}	-0.008	0.011	0.446
AD	rs139136389	19:45427136	T	C	-0.494	0.085	6.43×10^{-09}	0.076	0.050	0.129
AD	rs147711004	19:45337918	A	G	1.135	0.037	1.00×10^{-200}	0.035	0.028	0.207
AD	rs150685845	19:45675180	G	A	0.556	0.065	6.62×10^{-18}	-0.063	0.045	0.161
AD	rs1582763	11:60021948	A	G	-0.123	0.015	1.19×10^{-16}	-0.003	0.010	0.804

AD	rs34665982	6:32560306	C	T	-0.097	0.017	5.80×10^{-09}	0.011	0.012	0.381
AD	rs3740688	11:47380340	T	G	0.094	0.014	9.70×10^{-11}	0.005	0.009	0.605
AD	rs3851179	11:85868640	C	T	0.120	0.015	5.81×10^{-16}	0.019	0.010	0.049
AD	rs6733839	2:127892810	T	C	0.169	0.015	4.02×10^{-28}	0.018	0.011	0.097
AD	rs679515	1:207750568	C	T	-0.151	0.018	1.55×10^{-16}	0.002	0.011	0.871
AD	rs72654445	19:45417200	A	G	-0.543	0.081	2.27×10^{-11}	-0.019	0.049	0.693
AD	rs73223431	8:27219987	T	C	0.094	0.015	8.34×10^{-10}	0.009	0.010	0.380
AD	rs7412	19:45412079	T	C	-0.467	0.031	6.40×10^{-53}	0.004	0.016	0.779
AD	rs867230	8:27468503	A	C	0.133	0.016	3.49×10^{-17}	-0.003	0.010	0.768
AD	rs9381563	6:47432637	T	C	-0.082	0.015	2.93×10^{-08}	-0.008	0.011	0.462
PD	rs10451230	17:16035225	T	A	-0.096	0.018	4.42×10^{-08}	-0.006	0.009	0.557
PD	rs10513789	3:182760073	G	T	-0.160	0.022	3.18×10^{-13}	-0.021	0.011	0.061
PD	rs10847864	12:123326598	T	G	0.127	0.018	9.81×10^{-13}	0.010	0.010	0.336
PD	rs12934900	16:30923602	T	A	0.122	0.018	4.33×10^{-11}	-0.001	0.010	0.956
PD	rs144814361	10:121410917	T	C	0.441	0.068	9.07×10^{-11}	0.100	0.047	0.031
PD	rs329647	11:133764666	C	G	-0.113	0.018	1.94×10^{-10}	-0.002	0.011	0.831

PD	rs34311866	4:951947	C	T	0.227	0.023	7.97×10^{-23}	0.002	0.013	0.860
PD	rs35265698	6:32561334	G	C	-0.200	0.030	3.93×10^{-11}	0.007	0.013	0.596
PD	rs356203	4:90666041	T	C	-0.240	0.018	3.01×10^{-41}	0.004	0.010	0.693
PD	rs35749011	1:155135036	A	G	0.751	0.066	5.02×10^{-30}	0.050	0.042	0.228
PD	rs4488803	3:58218352	A	G	-0.114	0.020	1.08×10^{-08}	-0.008	0.009	0.424
PD	rs4588066	18:40672964	A	G	0.105	0.018	4.45×10^{-09}	-0.003	0.010	0.784
PD	rs4613239	2:169119609	G	C	0.178	0.025	6.21×10^{-13}	0.010	0.013	0.474
PD	rs4698412	4:15737348	A	G	0.126	0.017	7.05×10^{-14}	-0.003	0.010	0.798
PD	rs4774417	15:61993702	A	G	0.105	0.019	4.63×10^{-08}	0.000	0.011	0.975
PD	rs58879558	17:44095467	C	T	-0.238	0.025	1.36×10^{-21}	-0.017	0.012	0.170
PD	rs620490	8:16697579	G	T	-0.117	0.019	6.46×10^{-10}	0.004	0.010	0.706
PD	rs6741007	2:135537119	G	T	-0.123	0.018	2.09×10^{-12}	0.002	0.010	0.849
PD	rs75505347	12:40885549	T	C	0.392	0.067	6.12×10^{-09}	0.084	0.044	0.057
PD	rs75646569	5:60345424	G	T	0.192	0.027	5.62×10^{-13}	0.006	0.014	0.650
PD	rs7695720	4:77183300	C	A	-0.126	0.021	1.53×10^{-09}	-0.017	0.011	0.130
PD	rs823106	1:205656453	C	G	-0.149	0.024	4.10×10^{-10}	0.021	0.013	0.109

PD	rs858295	7:23245569	G	A	-0.104	0.018	3.83×10^{-09}	0.004	0.010	0.673
ALS	rs10463311	5:150410835	T	C	-0.085	0.016	4.00×10^{-08}	-0.008	0.010	0.460
ALS	rs12973192	19:17753239	G	C	0.121	0.015	3.92×10^{-15}	-0.001	0.011	0.907
ALS	rs142321490	12:58676132	C	G	0.317	0.051	6.15×10^{-10}	-0.014	0.040	0.733
ALS	rs3849943	9:27543382	T	C	-0.176	0.016	3.77×10^{-30}	0.007	0.011	0.504
ALS	rs74654358	12:64881967	A	G	0.198	0.034	4.66×10^{-09}	-0.024	0.027	0.387
ALS	rs75087725	21:45753117	A	C	0.515	0.067	1.85×10^{-14}	0.084	0.056	0.131

Note: SNP, Single-nucleotide polymorphism; EA, effect allele; OA, other allele; Beta (SE) represents coefficients by the additive regression model, and Beta > 0 denotes an additional copy of effect allele can increase serum GDF-15 levels or risks of AD/PD/ALS.

Source dataset of AD (ieu-b-2), PD (ieu-b-7) and ALS (ebi-a-GCST005647) were curated in "MR-base" and instrumental variables were directly retrieved (*P*-value threshold at 5×10^{-8}; clumping distance at 10 Mb; Linkage disequilibrium r^2 at 0.001), while corresponding summary statistics of GDF-15 were accessible from NIH Figshare repositories (https://github.com/rivas-lab/biomarkers).

Supplementary Table 4. Linkage disequilibrium matrix (*r*-value) utilized in the IVW method adjusted for correlated instrumental variables of GDF-15

	rs3195944_G_A	rs749451_T_C	rs17725099_A_G	rs888663_G_T	rs1227731_A_G
rs3195944_G_A	1				
rs749451_T_C	-0.241	1			
rs17725099_A_G	-0.077	-0.494	1		
rs888663_G_T	-0.179	0.545	-0.284	1	
rs1227731_A_G	0.874	-0.261	-0.082	-0.191	1

Note: Linkage disequilibrium statistics were calculated using "ld_matrix" (TwoSampleMR package, the European panel, 1000 Genomes Project Phase 3). The sign of *r*-value for a pair of variants indicated the relatedness of in-phase alleles. For instance, rs888663_G_T and rs749451_T_C were in linkage disequilibrium (r = 0.545), where G allele of rs888663 was correlated with T allele of rs749451. In the scenario of a negative sign, rs17725099_A_G and rs749451_T_C were in linkage disequilibrium (r = -0.494), where G allele of rs17725099 was correlated with T allele of rs749451.

Supplementary Table 5. Associations of GDF-15 with AD, PD and ALS in Mendelian randomization analyses by each instrumental variant and IVW adjusted for correlations

Outcomes	SNPs	OR	95% CI	*P*-value
AD	rs888663	1.04	0.91–1.19	0.558
AD	rs749451	1.10	0.95–1.28	0.211
AD	rs1227731	1.26	1.08–1.46	**0.003**
AD	rs3195944	1.25	1.06–1.48	**0.007**
AD	rs17725099	0.98	0.74–1.30	0.889
AD	**All - IVW**	1.14	1.04–1.24	**0.004**
AD	**IVW adjusted for correlation**	1.11	1.05–1.19	**0.001**
PD	rs888663	1.11	0.95–1.29	0.174
PD	rs749451	1.02	0.86–1.21	0.821
PD	rs1227731	0.96	0.81–1.12	0.584
PD	rs3195944	0.98	0.83–1.15	0.768
PD	rs17725099	1.09	0.79–1.50	0.603
PD	**All - IVW**	1.02	0.94–1.10	0.597

PD	**IVW adjusted for correlation**	1.03	0.96–1.10	0.440
ALS	rs888663	0.96	0.86–1.07	0.431
ALS	rs749451	0.94	0.83–1.06	0.310
ALS	rs1227731	0.95	0.84–1.08	0.455
ALS	rs3195944	0.97	0.86–1.10	0.645
ALS	rs17725099	0.95	0.76–1.20	0.687
ALS	**All - IVW**	0.96	0.90–1.01	0.120
ALS	**IVW adjusted for correlation**	0.95	0.91–1.00	0.155

Note: AD, Alzheimer's disease; ALS, amyotrophic lateral sclerosis; CI, confidence interval; IVW, inverse-variance weighted; OR, odds ratio; PD, Parkinson's disease; SNP, Single-nucleotide polymorphism.

Supplementary Table 6. Mendelian randomization sensitivity analyses examining horizontal pleiotropy

Exposures and outcomes	Number of SNPs	MR-Egger regression			Cochran's *Q* test		
		Intercept	Standard error	*P*-value	*Q*-statistic	I^2	*P*-value
GDF15 on AD	5	-0.045	0.038	0.323	5.9	32.5%	0.205
GDF15 on PD	5	0.017	0.037	0.683	2.3	0	0.685
GDF15 on ALS	5	-0.005	0.026	0.849	0.2	0	0.997
AD on GDF15	21	0.006	0.004	0.202	25.4	21.3%	0.186
PD on GDF15	23	-0.008	0.006	0.235	20.1	0	0.577
ALS on GDF15	6	-0.002	0.015	0.920	4.1	0	0.528

Note: I^2 statistic measured the percentage of variation across instrumental variants that is due to heterogeneity, $I^2 = (Q\text{-}df)/Q \times 100\%$, where df = N-1, df was degree of freedom, N was number of instrumental SNPs.

AD, Alzheimer's disease; ALS, amyotrophic lateral sclerosis; PD, Parkinson's disease; SNP, Single-nucleotide polymorphism.

Supplementary Figure Legend

Supplementary Figure 1. Pairwise statistics for linkage disequilibrium between instrumental variants for GDF-15. Linkage disequilibrium statistics were calculated using the European panel, 1000 Genomes Project Phase 3, in the web-based tool "LDlink" (https://ldlink.nci.nih.gov/#ldmatrix-tab). GDF15, Growth Differentiation Factor 15; PGPEP1, Pyroglutamyl-Peptidase I.

Supplementary Figure 2. Effects of genetically-predicted risk of AD, PD and ALS on circulating GDF-15 by Mendelian randomization analyses. AD, Alzheimer's disease; ALS, amyotrophic lateral sclerosis; CI, confidence interval; GDF15, growth differentiation factor 15; IVW, inverse-variance weighted model; PD, Parkinson's disease; SNP, single nucleotide polymorphism.

Supplementary Figure 3. Scatter plots showing effects of three neurodegenerative diseases on circulating GDF-15. AD, Alzheimer's disease; ALS, amyotrophic lateral sclerosis; GDF15, growth differentiation factor 15; IVW, inverse-variance weighted model; PD, Parkinson's disease; SNP, single nucleotide polymorphism.

Supplementary Figure 4. Leave-one-out plots in the Mendelian randomization sensitivity analyses of AD, PD and ALS on circulating GDF-15. AD, Alzheimer's disease; ALS, amyotrophic lateral sclerosis; GDF15, growth differentiation factor 15; IVW, inverse-variance weighted model; PD, Parkinson's disease.

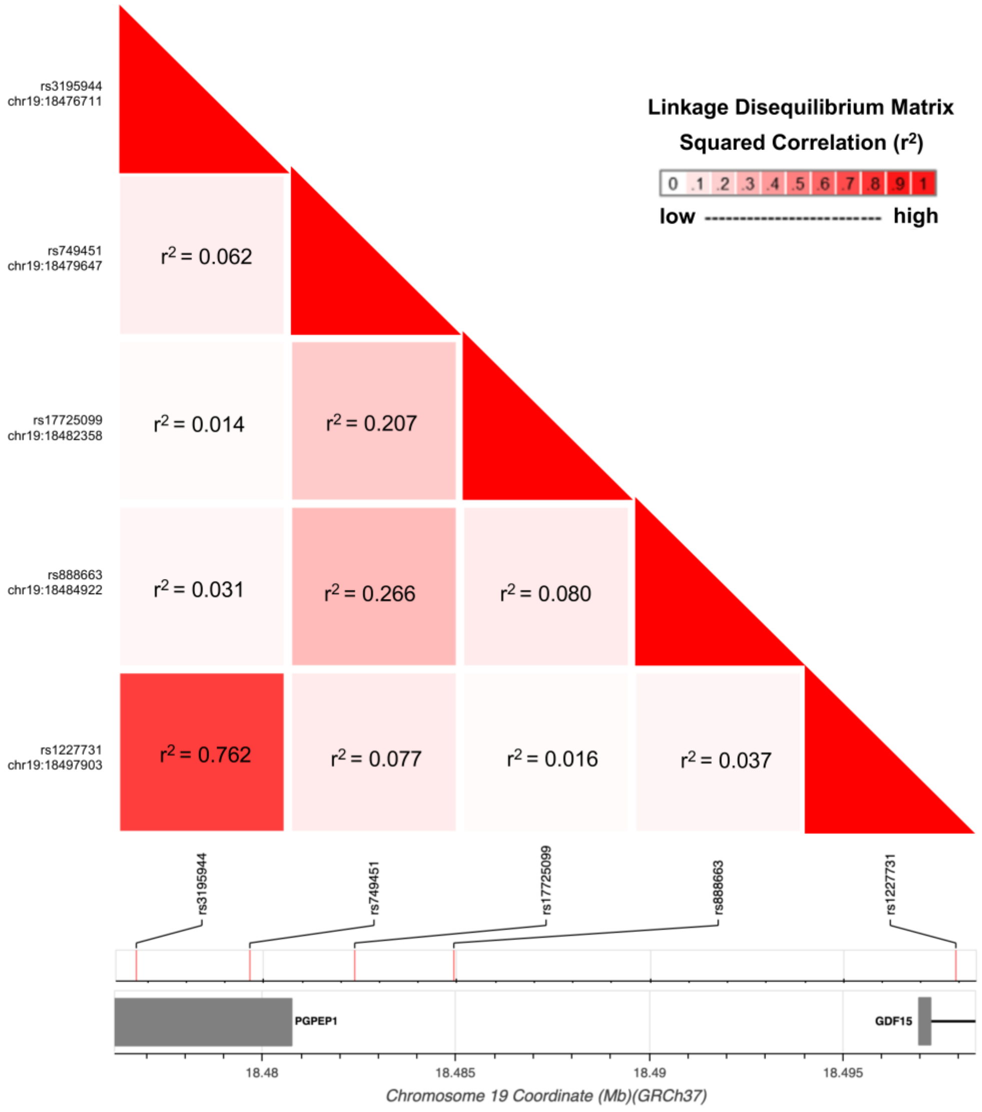

Supplementary Figure 1. Pairwise statistics for linkage disequilibrium between instrumental variants for GDF-15.

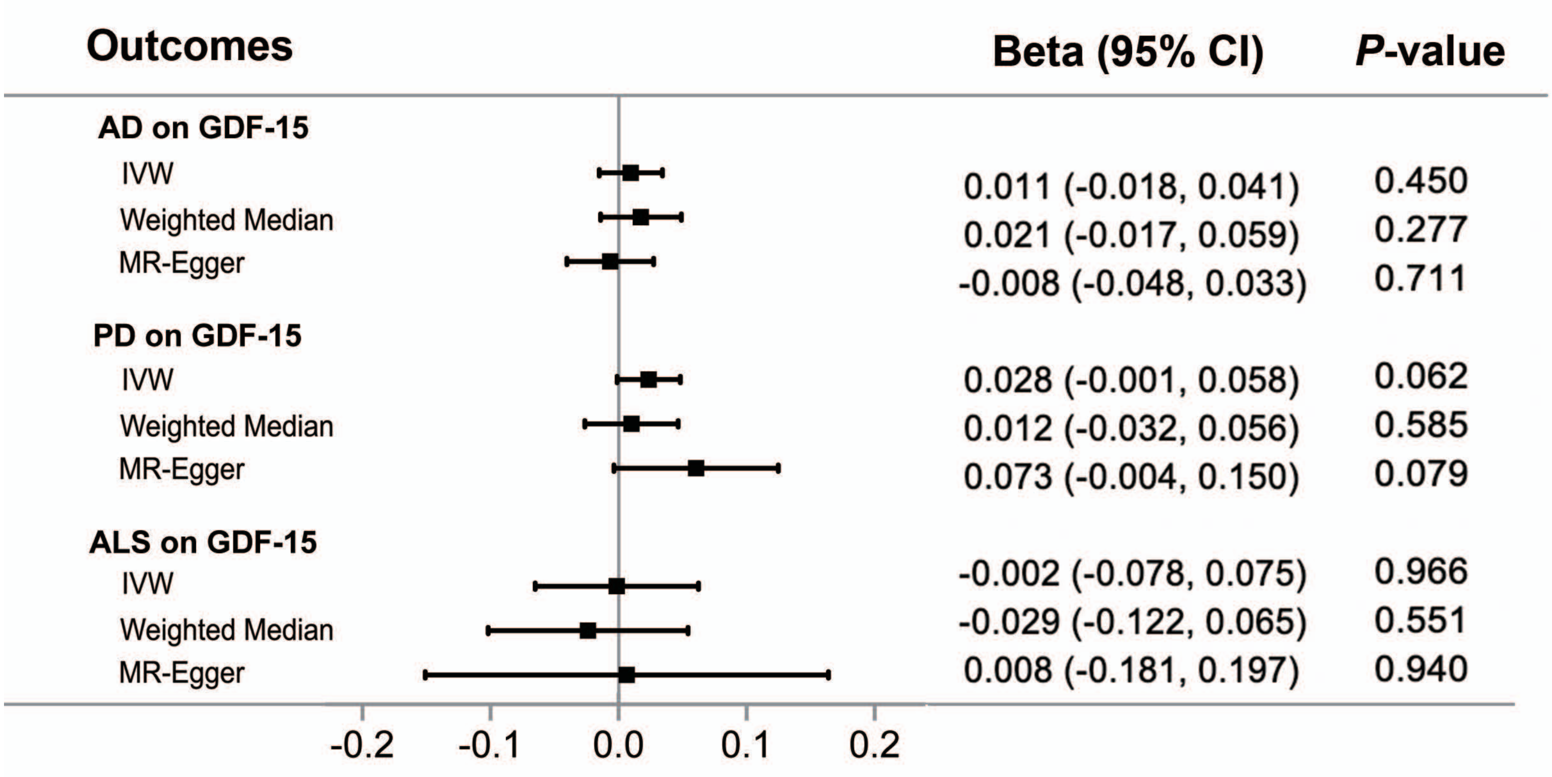

Supplementary Figure 2. Effects of genetically-predicted risk of AD, PD and ALS on circulating GDF-15 by Mendelian randomization analyses.

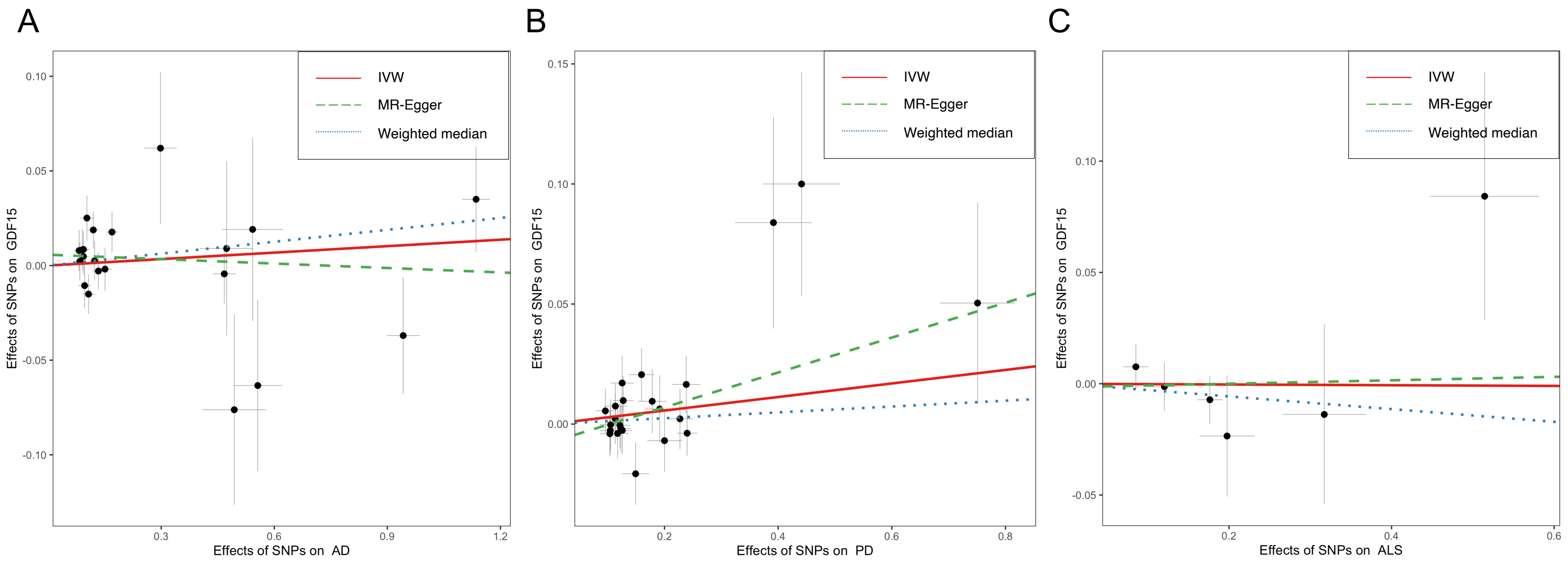

Supplementary Figure 3. Scatter plots showing effects of three neurodegenerative diseases on circulating GDF-15.

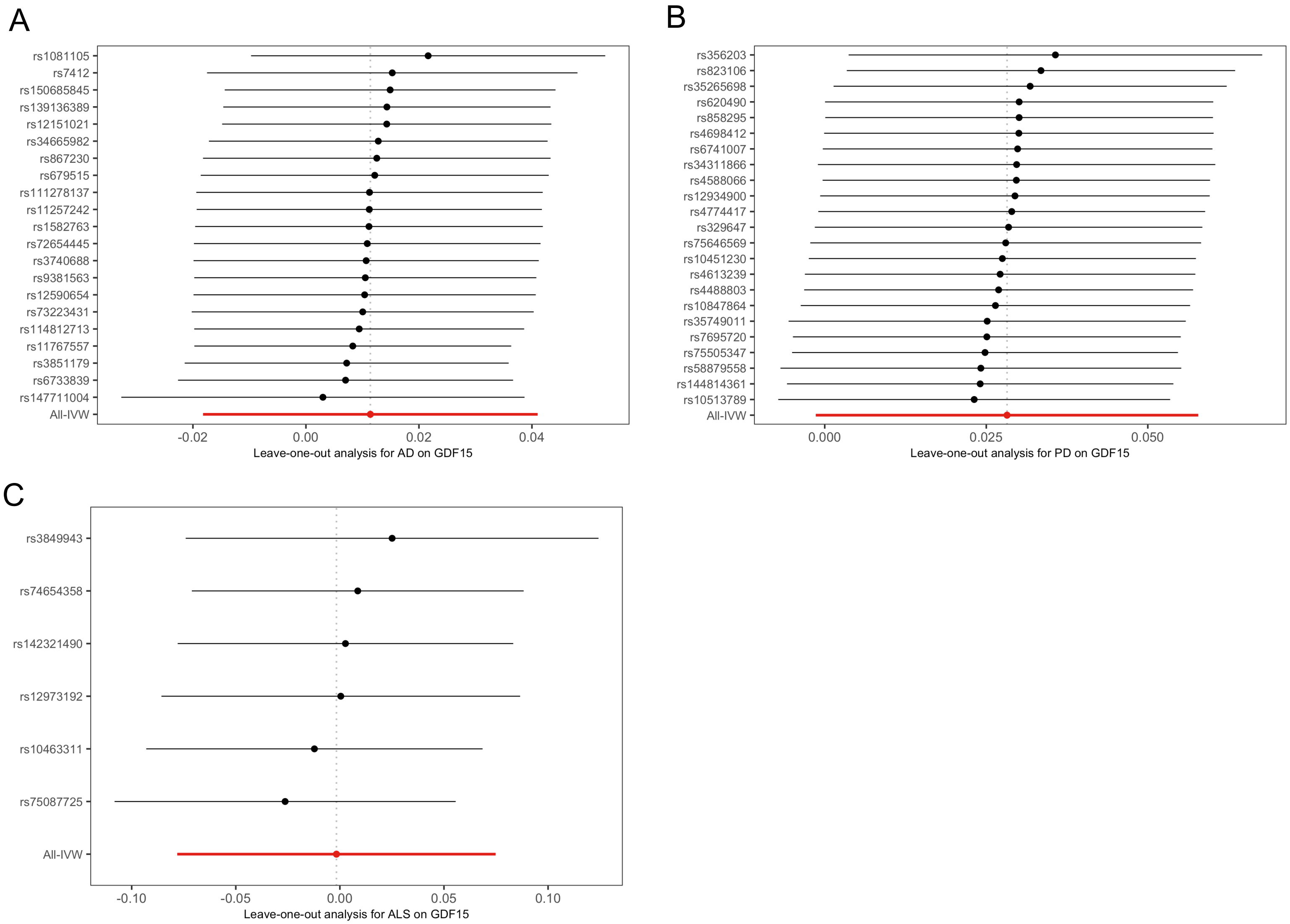

Supplementary Figure 4. Leave-one-out plots in the Mendelian randomization sensitivity analyses of AD, PD and ALS on circulating GDF-15.

Supplementary Texts

Statistical Analyses

The inverse-variance weighted (IVW) approach was used as the primary Mendelian randomization (MR) method (5). Given the SNP-exposure effect size X_k and its standard error σ_{X_k}, and the SNP-outcome statistics Y_k and σ_{Y_k}, the MR estimate can be derived by the Wald ratio $Y_k \ / \ X_k$ with its standard error $\sigma_{Y_k} \ / \ X_k$. Then the primary MR method, the inverse-variance weighted (IVW) model gave an overall estimate $\hat{\beta}_{MR}$ and its standard error $\hat{\sigma}_{MR}$ using formula:

$$\hat{\beta}_{IVW} = \sum X_k Y_k \sigma_{Y_k}^{-2} / \sum X_k^2 \sigma_{Y_k}^{-2}$$

$$\hat{\sigma}_{IVW} = \sqrt{1 \ / \sum X_k^2 \sigma_{Y_k}^{-2}}$$

In case of correlated instrumental variables, let $\rho_{k_1 k_2}$ be the linkage disequilibrium r-value for SNP_1 and SNP_2, with signs indicating the harmonization and relatedness of effect alleles and reference alleles, and the matrix was given by $\Omega_{k_1 k_2} = \sigma_{Y_{k_1}} \sigma_{Y_{k_2}} \rho_{k_1 k_2}$, then the extended IVW method (6) adjusted for the correlations between instrumental SNPs yielded an overall estimate:

$$\hat{\beta}_{IVW_{extension}} = (\Sigma X_k^T \Omega^{-1} X_k)^{-1} \Sigma X_k^T \Omega^{-1} Y_k$$

$$\hat{\sigma}_{IVW_{extension}} = \sqrt{\frac{\Sigma_{k_1} \Sigma_{k_2} \rho_{k_1 k_2} X_{k_1} X_{k_2} \sigma_{Y_{k_1}}^{-1} \sigma_{Y_{k_2}}^{-1}}{(\Sigma X_k^2 \sigma_{Y_k}^{-2})^2}}$$

References

(1) Jiang J, Thalamuthu A, Ho JE, et al. A Meta-Analysis of Genome-Wide Association Studies of Growth Differentiation Factor-15 Concentration in Blood. Front Genet. 2018;9:97.

(2) Kunkle BW, Grenier-Boley B, Sims R, et al. Genetic meta-analysis of diagnosed Alzheimer's disease identifies new risk loci and implicates Abeta, tau, immunity and lipid processing. Nat Genet. 2019 Mar:414-430.

(3) Nalls MA, Blauwendraat C, Vallerga CL, et al. Identification of novel risk loci, causal insights, and heritable risk for Parkinson's disease: a meta-analysis of genome-wide association studies. Lancet Neurol. 2019;18:1091-1102.

(4) Nicolas A, Kenna KP, Renton AE, et al. Genome-wide Analyses Identify KIF5A as a Novel ALS Gene. Neuron. 2018;97:1268-1283 e6.

(5) Burgess S, Butterworth A, Thompson SG. Mendelian randomization analysis with multiple genetic variants using summarized data. Genet Epidemiol. 2013; 37, 658-665

(6) Burgess S, Dudbridge F, Thompson SG. Combining information on multiple instrumental variables in Mendelian randomization: comparison of allele score and summarized data methods. Stat Med. 2016; 35,1880-906.
